# Efficacy and Safety of the Injection of the Traditional Chinese Medicine Puerarin for the Treatment of Diabetic Peripheral Neuropathy: A Systematic Review and Meta-Analysis of 53 Randomized Controlled Trials

**DOI:** 10.1155/2018/2834650

**Published:** 2018-01-24

**Authors:** Baocheng Xie, Qinghui Wang, Chenhui Zhou, Jiahuan Wu, Daohua Xu

**Affiliations:** ^1^Department of Pharmacology, Guangdong Medical University, Dongguan 523808, China; ^2^School of Nursing, Guangdong Medical University, Dongguan 523808, China; ^3^Institute of Traditional Chinese Medicine and New Pharmacy Development, Guangdong Medical University, Dongguan 523808, China

## Abstract

**Objective:**

The injection of the traditional Chinese patent medicine puerarin has been widely used in the treatment of various diseases such as angina pectoris or ischemic stroke. We aim to evaluate the efficacy and safety of puerarin injection for the treatment of diabetic peripheral neuropathy (DPN).

**Methods:**

A systematic literature search was performed in seven medical databases from their inception until June 2017. 53 studies with RCTs, totaling 3284 patients, were included in this meta-analysis. The included studies were assessed by the Cochrane risk of bias and analyzed by Review Manager 5.3 software.

**Results:**

The meta-analysis showed that puerarin injection for the treatment of DPN was significantly better compared with the control group in terms of the total effective rate. The result showed that puerarin injection for the treatment of DPN can significantly increase the probability of sensory nerve conduction velocity (SNCV) and motor nerve conduction velocity (MNCV) of the median and peroneal nerves.

**Conclusions:**

This meta-analysis demonstrated that puerarin injection may be more effective and safe for the treatment of DPN. However, further and higher quality RCTs are required to prove its efficacy and provide meaningful evidence for clinical treatment due to the poor methodological quality.

## 1. Introduction

Diabetic peripheral neuropathy (DPN) is one of the most common neuropathies of diabetes mellitus (DM) and can lead to foot ulceration and amputation [1]. It affects sensory, autonomic, and motor nerve functions [2]. The annual costs of DPN and its complications are 10.9 billion dollars with a cost-of-illness model in the United States [3]. The enormous therapeutic costs, pain interference with function, and disabilities lead to a significant impact on the quality of life (QOL) and life expectancy of DPN [4]. Available treatments can only ease symptoms and there is currently no effective treatment reversing the progression of DPN [5]. Western medicines such as methylcobalamin and neurotrophin are usually used in the treatment of DPN. But the therapeutic effect of the western medicine was poor in patients with DPN. The traditional Chinese patent medicine puerarin is one of the flavonoids extracted from Gegen and pharmacological studies have confirmed that puerarin injection can lower blood sugar, significantly improve the microcirculation, expand the coronary arteries, and reduce platelet aggregation. Puerarin injection has shown certain advantages in the treatment of DPN and has been widely used for more than 20 years in China. Dysfunction and damage of myelinated and unmyelinated fibers can lead to the symptoms of painful neuropathy, ulceration, and demyelination and axonal degeneration has been considered to be the sign of the pathology of human diabetic neuropathy [6]. Puerarin injection for the treatment of DPN could significantly increase the probability of sensory nerve conduction velocity and motor nerve conduction velocity [7]. However, the use of puerarin injection in the treatment of DPN in other countries is not high and the clinical efficacy of puerarin injection combined with some western medicine was not certain. Therefore, our study included 53 RCTs with a total of 3284 patients who were included in order to acquire high-quality evidence for the clinical efficacy and safety of puerarin injection in DPN, and we also performed subgroup analyses in order to timely find out the clinical efficacy of puerarin injection combined with western medicine in the treatment of DPN.

## 2. Methods

### 2.1. Literature Search

We searched clinical studies databases, including CBM, CNKI, PubMed, Embase, ClinicalTrials.gov, and Cochrane Central Register of Controlled Trials, from their inception until June 2017. We used the following search terms: (1) “Puerarin”, “Puerarin injection”, “Kakkonein injection” connected with “OR”; (2) “Diabetic peripheral neuropathy”, “peripheral neuropathy”, “Diabetic”, “diabetic complication” connected with “OR”; (3) “randomized controlled” or “Clinical Trials”. Then, the above search terms of (1), (2), and (3) were connected with “AND”. We manually searched the references of the original and review articles for possible related studies.

### 2.2. Study Selection

For the systematic review, we searched 53 clinical studies that met the following criteria: (1) studies including patients with DPN, (2) studies including patients who received puerarin injection therapy, (3) studies reported as RCTs, (4) studies where the control group received standard therapy or recovery treatment, and (5) studies that reported efficacy and safety issues.

### 2.3. Data Extraction and Quality Assessment

Two of the authors independently extracted the data of the literature and carried out a quality assessment process according to the predefined inclusion criteria. Differences between the two authors were resolved by discussion with the third author. We used the Cochrane risk of bias tool for the quality evaluation of the RCTs. This quality evaluating strategy included criteria concerning aspects of random sequence generation, allocation concealment, blinding of participants and personnel, blinding of outcome assessors, incomplete outcome data, selective reporting, and other bias.

### 2.4. Statistical Analyses

In this meta-analysis, all statistical analyses were performed using RevMan software version 5.3 and we used RR with 95% CI for the analyses of dichotomous data, whereas the continuous data were presented as MD or SWD with 95% CI. Heterogeneity between the studies was determined using the chi-square test, with the *I*^2^ statistic, where *I*^2^ < 25% represents mild inconsistency, values between 25% and 50% represent moderate inconsistency, and values > 50% suggest severe heterogeneity between the studies. We defined *I*^2^ > 50% as an indicator of significant heterogeneity among the trials. We used random-effects models to estimate the pooled results to minimize the influence of potential clinical heterogeneity among the studies and the statistical significance was assumed at *P* < 0.05. Subgroup analyses were assessed using the *χ*^2^ test. Sensitivity analyses were performed to evaluate the robustness of merged results, by removing individual studies. Publication bias was assessed by means of funnel plots.

## 3. Results

### 3.1. Search Results

**Figure 1:**
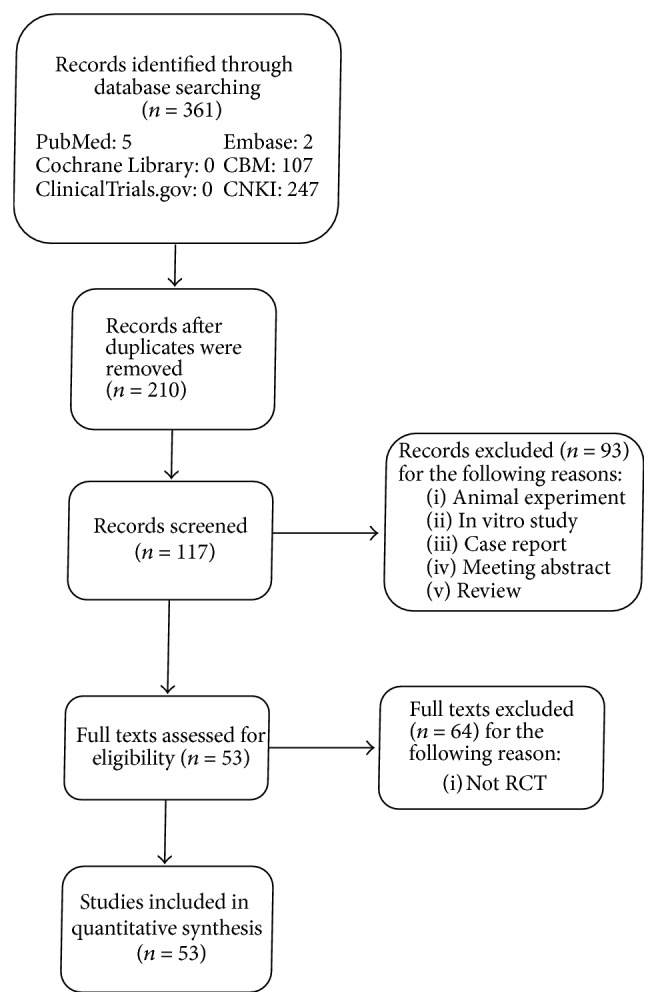
Flow chart and strategy of the meta-analysis.

A systematic search of studies published until June 2017 was performed through CBM, CNKI, PubMed, Embase, ClinicalTrials.gov, and Cochrane Central Register of Controlled Trials databases since their inception. A total of 361 literatures were searched and 53 studies were included in the inclusion criteria; the literature search procedure is shown in Figure 1.

### 3.2. Study Characteristics

The general characteristics of the included studies are listed in Table 1. The included studies were 53 RCTs with a total of 3284 patients: the treatment group of puerarin injection combined with mecobalamin and the control group with mecobalamin (13 studies); the treatment group of puerarin injection combined with epalrestat and the control group with epalrestat (3 studies); the treatment group of puerarin injection combined with danshen injection and the control group with danshen injection (5 studies); the treatment group of puerarin injection combined with vitamins B_1_ and B_12_ and the control group with vitamins B_1_ and B_12_ (4 studies).

### 3.3. Quality Assessment

**Figure 2:**
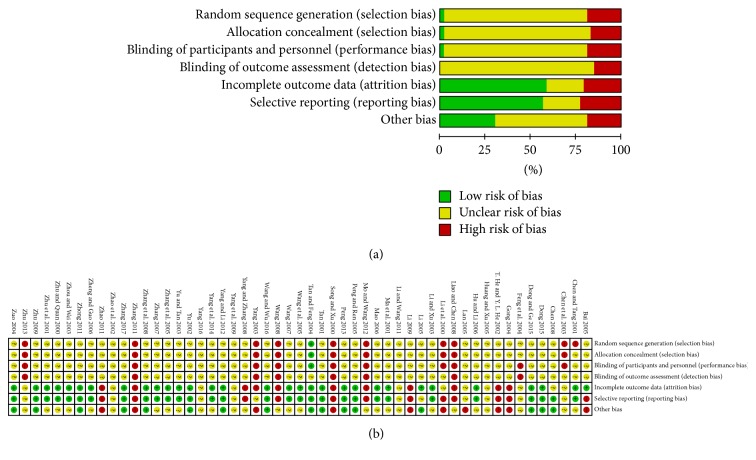
Quality of RCTs according to the Cochrane Collaboration Manual. (a) Summary of RCTs quality showing the percentage of RCTs satisfying each risk of bias graph. (b) Detailed item-by-item analysis of the risk of bias summary.

The risks of bias in the included studies were evaluated by the Cochrane assessment tool and these results are summarized in Figure 2. One study was at low risk of bias for random sequence and reported the details of allocation concealment. Forty-six studies were at an unclear risk of bias for blinding of participants and personnel according to the Cochrane collaboration tool. Thirty-one studies reported methods with a low risk of attrition bias and thirty studies reported a low risk of reporting bias.

### 3.4. Major Outcomes

#### 3.4.1. The Total Effective Rate

**Figure 3:**
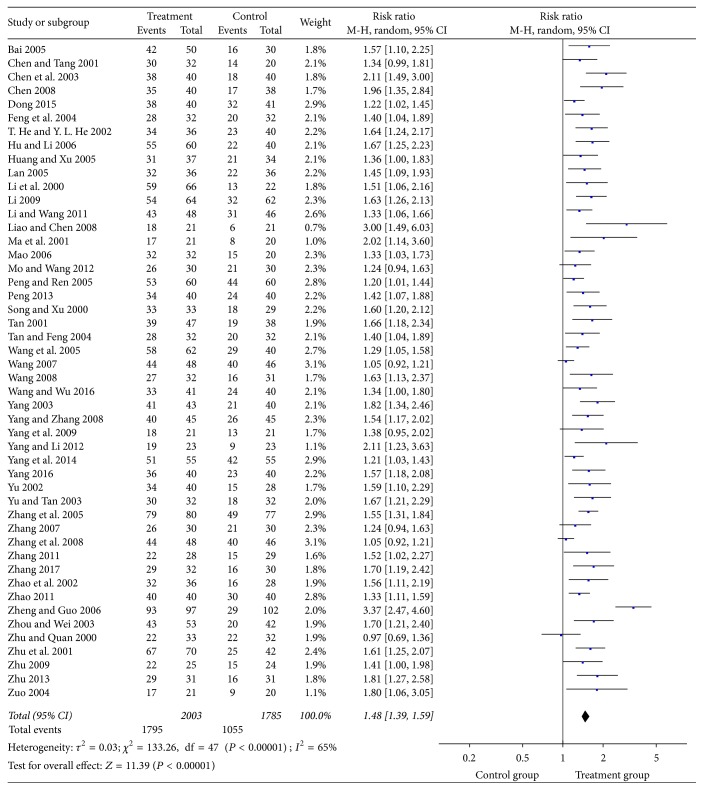
Forest plot of the meta-analysis with the total effective rate.

The total effective rate was reported in 48 studies with a total of 3798 patients treated with puerarin injection and 2840 patients in the control group. The meta-analysis showed that puerarin injection for the treatment of DPN was significantly better compared with the control group in terms of the total effective rate (RR = 1.48, 95% CI = 1.39–1.59, *P* < 0.00001) (Figure 3).

#### 3.4.2. Sensory Nerve Conduction Velocity

**Figure 4:**
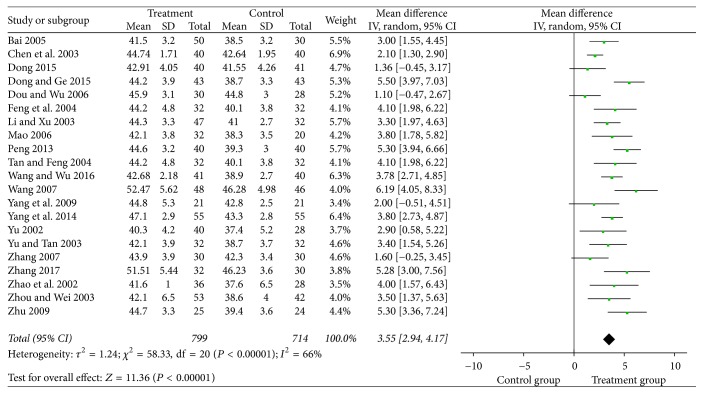
Forest plot of the meta-analysis with SNCV of the median nerve.

**Figure 5:**
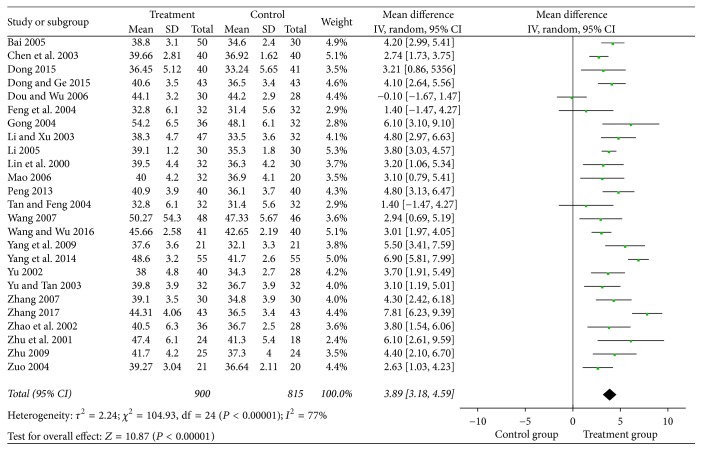
Forest plot of the meta-analysis with SNCV of the peroneal nerve.

In 21 studies, the median nerve was included in the analysis and the results indicated that puerarin injection significantly increased the sensory nerve conduction velocity of the median nerve (MD = 3.55, 95% CI = 2.94–4.17, *P* < 0.00001) compared with the control group (Figure 4). The peroneal nerve was reported in 25 studies with a total of 900 patients treated with puerarin injection and 815 patients in the control group. The results showed that puerarin injection for the treatment of DPN can significantly increase the sensory nerve conduction velocity of the peroneal nerve (MD = 3.89, 95% CI = 3.18–4.59, *P* < 0.00001) (Figure 5).

#### 3.4.3. Motor Nerve Conduction Velocity

**Figure 6:**
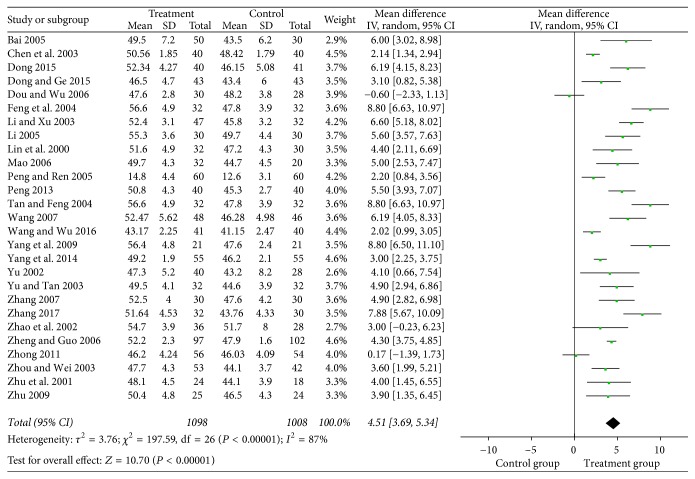
Forest plot of the meta-analysis with MNCV of the median nerve.

**Figure 7:**
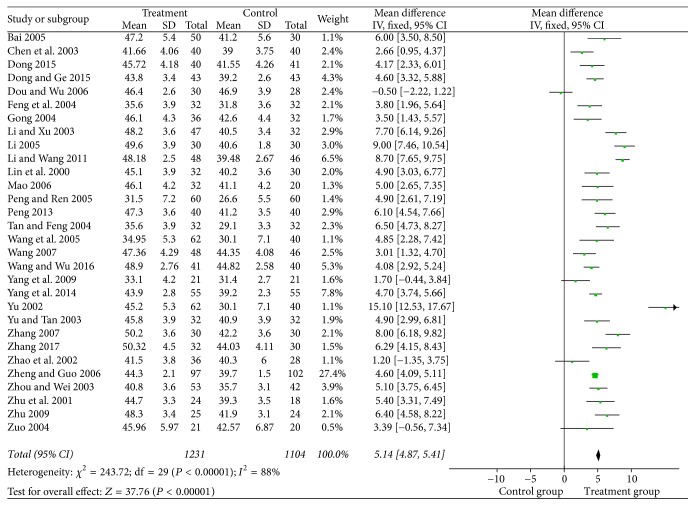
Forest plot of the meta-analysis with MNCV of the peroneal nerve.

There were 27 studies with a total of 2106 patients in regard to peroneal nerve and 29 studies with a total of 2233 patients in regard to median nerve. Results of analysis indicated that puerarin injection for the treatment of DPN can significantly increase motor nerve conduction velocity of the median nerve (MD = 4.51, 95% CI = 3.69–5.43, *P* < 0.00001) and peroneal nerve (MD = 5.14, 95% CI = 4.87–5.41, *P* < 0.00001) compared with the control group (Figures 6 and 7).

### 3.5. Subgroup Analysis

#### 3.5.1. Puerarin Injection + Mecobalamin versus Mecobalamin

Patients with DPN were treated with puerarin injection and mecobalamin in the treatment group and with mecobalamin in the control group. The results of subgroup analysis showed that puerarin injection combined with mecobalamin therapy was more effective than mecobalamin in the total effective rate (RR = 1.31, 95% CI = 1.22–1.41, *P* < 0.00001), SNCV of the median nerve (MD = 3.64, 95% CI = 2.78–4.5, *P* < 0.0001), SNCV of the peroneal nerve (MD = 4.26, 95% CI = 2.98–5.55, *P* < 0.00001), MNCV of the median nerve (MD = 5.18, 95% CI = 3.51–6.85, *P* < 0.00001), and MNCV of the peroneal nerve (MD = 4.54, 95% CI = 3.23–5.85, *P* < 0.00001) (Table 2).

#### 3.5.2. Puerarin Injection + Vitamins B_1_ and B_12_ versus Vitamins B_1_ and B_12_

Patients with DPN were treated with puerarin injection and vitamins B_1_ and B_12_ in the treatment group and with vitamins B_1_ and B_12_ in the control group. The results of subgroup analysis showed that puerarin injection combined with vitamins B_1_ and B_12_ therapy was better than vitamins B_1_ and B_12_ in the total effective rate (RR = 1.61, 95% CI = 1.24–2.10, *P* = 0.0004), SNCV of the median nerve (MD = 5.43, 95% CI = 4.16–6.7, *P* < 0.00001), SNCV of the peroneal nerve (MD = 3.96, 95% CI = 2.94–4.97, *P* < 0.00001), MNCV of the median nerve (MD = 5.14, 95% CI = 2.31–7.97, *P* = 0.0004), and MNCV of the peroneal nerve (MD = 5.01, 95% CI = 4.06–5.95, *P* < 0.00001) (Table 2).

#### 3.5.3. Puerarin Injection + Epalrestat versus Epalrestat

Patients with DPN were treated with puerarin injection and epalrestat in the treatment group and with epalrestat in the control group. The results of subgroup analysis showed that puerarin injection combined with epalrestat therapy could significantly improve the total effective rate (RR = 1.38, 95% CI = 1.12–1.69, *P* = 0.002) and increase the SNCV of the median nerve (MD = 3.43, 95% CI = 1.28–5.58, *P* = 0.002) and peroneal nerve (MD = 2.57, 95% CI = 0.08–5.06, *P* = 0.04) (Table 2).

#### 3.5.4. Puerarin Injection versus Danshen Injection

Patients with DPN were treated with puerarin injection in the treatment group and with danshen injection in the control group. The results of subgroup analysis showed that puerarin injection therapy was more effective than danshen injection in the total effective rate (RR = 1.44, 95% CI = 1.24–1.68, *P* < 0.00001) and the SNCV of the peroneal nerve (MD = 3.10, 95% CI = 1.91–4.29, *P* < 0.00001) (Table 2).

### 3.6. Heterogeneity and Publication Bias

**Figure 8:**
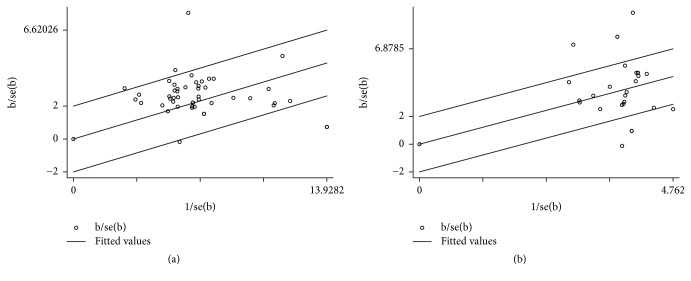
Meta-analysis of sensitivity. (a) Galbraith plot of the total effective rate. (b) Galbraith plot of SNCV of the peroneal nerve.

**Figure 9:**
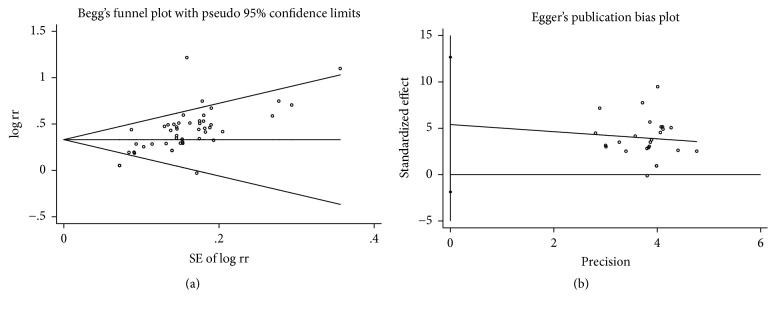
Meta-analysis of publication bias. (a) Funnel plot of the total effective rate. (b) Funnel plot of SNCV of the peroneal nerve.

According to this meta-analysis, sensitivity analysis was performed using Galbraith plot for the total effective rate and SNCV of the peroneal nerve. The results showed that there was no substantial change in the total effective rate, indicating that the results of the meta-analysis were credible. But a significant heterogeneity was noted for SNCV of the peroneal nerve using the random-effects model (*I*^2^ > 50%) (Figure 8). A significant symmetry was noted for distribution in funnel plots of the total effective rate. The quantitation of Egger's test with SNCV of the peroneal nerve (*P* > 0.138, 95% CI = −1.86–12.6) indicated that publication bias was not obvious in the included studies (Figure 9).

### 3.7. Safety

In the 53 included studies, two studies reported that 8 patients had dizziness after injection in the puerarin group and the dizziness began to ease up after slowing down the intravenous infusion. One study reported that 2 patients felt facial fever and the other patients did not experience any other adverse drug reactions. There were 1 patient with nausea and 1 patient with diarrhea in the treatment group and 2 patients with nausea in the control group. No severe adverse drug reaction occurred in the treatment group and control group.

## 4. Discussion

### 4.1. Main Outcome

Puerarin is an isoflavone compound and it is the main active ingredient of* Pueraria lobata*. The pharmacological effects of puerarin can expand blood vessels, relieve vasospasm, and improve circulation. Puerarin injection as a traditional Chinese patent medicine has been widely used in the treatment of various diseases such as diabetic peripheral neuropathy (DPN), cardiovascular diseases, sudden deafness, angina pectoris, or ischemic stroke [18, 43, 44]. Our study included 53 RCTs with a total of 3284 patients who were included in order to acquire high-quality evidence for the clinical efficacy and safety of puerarin injection therapy in DPN. The result showed that puerarin injection for the treatment of DPN significantly improved the probability effect of total effective rate by 48% compared with control groups. Analyses of SNCV showed that puerarin injection for the treatment of DPN can significantly increase the conduction velocity of the median nerve and peroneal nerve by 3 m/s (*P* < 0.01). Analyses of MNCV demonstrated a significant improvement in the median nerve and peroneal nerve by 4 m/s (*P* < 0.01). The EMG showed that nerve conduction velocity increased by 1–5 m/s after treatment with puerarin injection.

### 4.2. Subgroup Analysis

Mecobalamin is one of the coenzyme forms of vitamin B_12_, which can promote the synthesis of lecithin and the formation of neuronal myelin in the body. Moreover, mecobalamin can promote neuronal differentiation and replication [61, 62]. It was reported that mecobalamin could improve neuropathic symptoms. In our meta-analysis of subgroup analysis, the results showed that puerarin injection combined with mecobalamin therapy was more effective than mecobalamin in the total effective rate, SNCV of the median nerve and peroneal nerve, and MNCV of the median nerve and peroneal nerve. We analyzed the effect of puerarin injection and vitamins B_1_ and B_12_ in the treatment group and vitamins B_1_ and B_12_ in the control group. The results of subgroup analysis showed that puerarin injection combined with vitamins B_1_ and B_12_ therapy was better than vitamins B_1_ and B_12_ in the total effective rate, SNCV of the median nerve and peroneal nerve, and MNCV of the median nerve and peroneal nerve.

Epalrestat is a noncompetitive and reversible aldose reductase inhibitor used for the treatment of diabetic neuropathy by relieving oxidative stress and suppressing the polyol pathway [63, 64]. A study [65] reported that 2190 patients were treated with epalrestat, and the result showed that the improvement rate of the subjective symptoms was 75% and that of the nerve function test was 36%. We performed a meta-analysis on patients with DPN treated with puerarin injection combined with epalrestat in the treatment group and with epalrestat in the control group. The results of subgroup analysis showed that puerarin injection combined with epalrestat therapy could significantly improve the total effective rate by 38% and increase the SNCV of the median nerve and peroneal nerve, compared with the control group.

According to the above analysis, the clinical efficacy of puerarin injection combined with western medicine was significantly better than that of western medicine in the treatment of DPN.

### 4.3. Limitations and Critical Considerations

We must be tapered in view of the limitations of this meta-analysis with low quality, high heterogeneity, and publication bias. The study would lead to publication bias because of low quality trials, such as a lack of reporting about random sequence generation and concealment, especially in early and small trials. The review includes 53 RCTs, with 3284 patients, which were published in Chinese. Most of the studies were just referring to randomized trials, but there were no specific randomized trials of random sequence generation, allocation concealment, and blinding of outcome assessment. The methodological quality was generally low in most of the studies, which perhaps led to a risk of bias. Sensitivity analysis was performed using Galbraith plot and the results showed that there was no substantial change in the total effective rate, indicating that the results of the meta-analysis were credible. But a significant heterogeneity was noted for SNCV of the peroneal nerve and median nerve using the random-effects model (*I*^2^ > 50%). We considered high heterogeneity among studies as studies differed in design, underlying disease, follow-up duration, and the drugs of treatment. Reporting bias is an important issue of meta-analysis. Many results of negative studies may be filtered or hidden in such a way that the studies become positive and some negative studies would be unpublished. Symmetry was noted for distribution in funnel plots of the total effective rate. The quantitation of Egger's test with SNCV of the peroneal nerve (*P* > 0.138) indicated that publication bias was not obvious in the included studies.

## 5. Conclusions

In summary, this systematic review and meta-analysis demonstrated that puerarin injection may be effective and safe for the treatment of DPN. Subgroup analyses indicated that the clinical efficacy of puerarin injection combined with western medicines such as mecobalamin and epalrestat was significantly better than that of western medicine in the treatment of DPN. However, further and higher quality RCTs are required to prove its efficacy and provide meaningful evidence for clinical treatment due to the poor methodological quality and lack of adequate safety data.

## Figures and Tables

**Table 1:** Characteristics of included articles.

Study	Sample (T/C)	Mean (T/C)	Intervention		Course of disease		Follow-up	Evaluation
			T	C	T	C		
Zheng and Guo 2006 [8]	61.7/63.2	97/102	Pue (Ivgtt) + scopolamine (IV)	Vit B_12_ (IM)	T2DM (6.7 y)		4 w	①④⑤
Wang et al. 2005 [9]	48	62/40	Pue (Ivgtt) + Mec (Ivgtt)	Mec (Ivgtt)	T2DM + DPN (1–4 m)		2 w	①⑤
Huang and Xu 2005 [10]	53/50	37/34	Pue (Ivgtt)	Anisodamine (Ivgtt)	T2DM (7.6 y) + DPN (2.3 y)	T2DM (8.3 y) + DPN (2.1 y)	20 d	①
Yu 2002 [11]	56.2/57.2	40/28	Pue (Ivgtt) + anisodamine (Ivgtt)	Danshen (Ivgtt)	T2DM (11.4 y) + DPN (7.6 y)	T2DM (12 y) + DPN (7.3 y)	30 d	①②③④⑤
Tan and Feng 2004 [12]	58.2/56.3	32/32	Pue (Ivgtt) + Vit B_1_, B_6_ (Ivgtt)	Vit B_1_, B_6_ (Ivgtt)	DM (10.3 y) + DPN (4.3 y)	DM (11.3 y) + DPN (3.5 y)	4 w	①②③④⑤
Yang 2003 [13]	Unclear	43/40	Pue (Ivgtt) + Mec (IM)	Vit B_12_ (IM)	Unclear	Unclear	12 d	①
Li 2009 [14]	55/55	64/62	Pue (Ivgtt) + nimodipine (Ivgtt)	Nimodipine (Ivgtt)	DM (3–21 y) + DPN (4 m–6 y)	DM (3–25 y) + DPN (3 m–5 y)	4 w	①
Wang 2008 [15]	55/55.5	32/31	Pue (Ivgtt) + nimodipine (Ivgtt)	Nimodipine (Ivgtt)	DM (5–24 y) + DPN (4 m–6 y)	DM (2–25 y) + DPN (3 m–6 y)	4 w	①
Zhao 2011 [16]	70.5/38–79	40/40	Pue (Ivgtt) + Mec (Po)	Mec (Po)	DM (6.5 y) + DPN (4.2 y)	DM (2–24 y) + DPN (1–17 y)	8 w	①
Zhao and Du 2003 [17]	58.8/No	36/28	Pue (Ivgtt) + Mec (Ivgtt)	Mec (Ivgtt)	DM (6–20 y) + DPN (1–11 y)	Unclear	6 w	①②③④⑤
Yang et al. 2014 [18]	56/56	29/55	Pue (Ivgtt) + Mec (Ivgtt)	Mec (Ivgtt)	DM + DPN (9.6 y)	DM + DPN (9.5 y)	4 w	①②③④⑤
Chen 2008 [19]	55/53.5	40/38	Pue (Ivgtt) + alprostadil (Ivgtt)	Alprostadil (Ivgtt)	Unclear	Unclear	6 w	①
Zhang et al. 2005 [20]	57.3	80/77	Pue (Ivgtt) + prostaglandin E_1_ (Ivgtt)	Prostaglandin E1 (Ivgtt)	Unclear	Unclear	14 d	①
Wang and Wu 2016 [21]	62.9/63.2	41/40	Pue (Ivgtt) + Epa (Po)	Epa (Po)	T2DM + DPN (12.7 y)	T2DM + DPN (13.0 y)	2 w	①②③④⑤
Yu and Tan 2003 [22]	58.2/58.8	32/32	Pue (Ivgtt) + Mec (IM)	Mec (Ivgtt)	DM (2–10 y) + DPN (5–56 m)	DM (2.5–11.5 y) + DPN (4–50 m)	20 d	①②③④⑤
Li and Xu 2003 [23]	57.2/58.3	47/32	Pue (Ivgtt) + Mec (Ivgtt)	Mec (Po)	DM (10.7 y) + DPN (3.5 y)	DM (8.8 y) + DPN (3.1 y)	4 w	①②③④⑤
Li and Wang 2011 [24]	47/47	48/46	Pue (Ivgtt) + Mec (Ivgtt)	Mec (Ivgtt)	DM (6 m–15 y) + DPN (6 m–5 y)	DM (8–15 y) + DPN (7–5 y)	6 w	①③⑤
Lan 2005 [25]	65.5/64.8	36/36	Pue (Ivgtt) + Mec (Ivgtt)	Danshen (Ivgtt)	DM + DPN (3 y)	DM + DPN (3.1 y)	6 w	①
Gong 2004 [26]	53.2/52.7	36/32	Pue (Ivgtt) + Mec (IM)	Mec (IM)	T2DM (10.2 y) + DPN (2.6 y)	T2DM (9.7 y) + DPN (2.7 y)	8 w	③⑤
Dou and Wu 2006 [27]	58.5/57.2	28/30	Pue (Ivgtt) + Epa (Po)	Epa (Po)	DM (6.4 y) + DPN (3.2 y)	DM (5.2 y) + DPN (3.0 y)	4 w	①②③④⑤
Peng 2013 [28]	56.2/57.9	40/40	Pue (Ivgtt) + Epa (Po)	Epa (Po)	T2DM (2–17 y) + DPN (4.1 y)	T2DM (2–18 y) + DPN (4.9 y)	4 w	①②③④⑤
Zhou and Wei 2003 [29]	35–75	53/42	Pue (Ivgtt)	Vit B_1_, B_12_ (IM)	Unclear	Unclear	30 d	①②④⑤
Chen et al. 2003 [30]	57.1/57	40/40	Pue (Ivgtt)	Vit B_1_, B_12_ (IM)	Unclear	Unclear	4 w	①②③④⑤
Liao and Chen 2008 [31]	64	21/21	Pue (Ivgtt)	Vit B_1_, B_12_ (IM)	Unclear	Unclear	2 w	①
Ma et al. 2001 [32]	58/55	21/20	Pue (Ivgtt)	Vit B_1_ (Po) + Vit B_12_ (IM)	DM + DPN (13 y)	DM + DPN (15 y)	15 d	①
Zhu 2013 [33]	61.3	31/31	Pue (Ivgtt)	Vit B	Unclear	Unclear	4 w	①
Chen and Tang 2001 [34]	56/54	32/20	Pue (Ivgtt) + Vit B_1_, B_6_ (IM)	Vit B_1_, B_12_ (IM)	DM (8.6 y) + DPN (7.2 y)	DM (9 y) + DPN (8 y)	20 d	①
Yang 2010 [35]	58.6/58.4	21/21	Pue (Ivgtt) + Mec (Po)	Mec (Po)	Unclear	Unclear	1 m	①②④⑤
Zhu 2009 [36]	59.6/60.3	25/24	Pue (Ivgtt) + *α*-lipoic acid (Ivgtt)	Pue (Ivgtt)	T2DM (11.9 y)	T2DM (11.6 y)	2 w	①②③④⑤
Zhu et al. 2001 [37]	54/56	50/42	Pue (Ivgtt)	Vit B_12_ (I.M.)	DM (144 m) + DPN (28.3 m)	DM (132 m) + DPN (30.4 m)	20 d	①②③④
Zhong 2011 [38]	55/54	65/54	Sodium ozagrel (Ivgtt) + Pue (Ivgtt)	Vit B_1_, B_12_ (IM)	DM (4 y)	DM (3.9)	14 d	②④
Mo and Wang 2012 [39]	65.4/66.2	30/30	Pue (Ivgtt) + Mec (Po)	Pue (Ivgtt)	Unclear	Unclear	2 w	①
Zhang 2007 [40]	62/60	30/30	Pue (Ivgtt) + Mec (IM)	Mec (I.M.)	T2DM (12.3 y) + DPN (4.3 y)	T2DM (11.6 y) + DPN (3.9 y)	4 W	①②③④⑤
Yang and Zhang 2008 [41]	58.6/59.6	45/45	Pue (Ivgtt) + Mec (Po)	Mec (Po)	T2DM (9.7 y)	T2DM (10.0 y)	4 w	①
Yang and Li 2012 [42]	57.2	23/23	Pue (Ivgtt)	Conventional therapy	Unclear	Unclear	2 w	①
Dong and Ge 2015 [43]	46.4/45.6	43/43	Pue (Ivgtt) + Vit B_1_, B_12_	Vit B_1_, B_12_	Unclear	Unclear	4 w	②③④⑤
Zhang 2017 [44]	61/59.4	32/30	Pue (Ivgtt) + Vit B_1_, B_12_ (IM)	Vit B_1_, B_12_ (IM)	Unclear	Unclear	14 d	①②③④⑤
Lin et al. 2000 [45]	58.4/56.9	66/22	Pue (Ivgtt) + Vit B_1_, B_12_ (IM)	Mec (Po) + Vit B_1_, B_12_ (IM)	DM (12.8 y) + DPN (4.2 y)	DM (11.6 y) + DPN (3.9 y)	60 d	①②③④⑤
Tan 2001 [46]	55.6/56.2	47/38	Pue (Ivgtt) + Vit B	Vit B	DM (1–21 y) + DPN (1 m–10 y)	DM (1–20 y) + DPN (1 m–8 y)	60 d	①
Zhu and Quan 2000 [47]	Unclear	33/32	Pue (Ivgtt)	Danshen (Ivgtt)	Unclear	Unclear	3 w	①
T. He and Y. L. He 2002 [48]	56	36/40	Pue (Ivgtt)	Danshen (Ivgtt)	Unclear	Unclear	6 w	①
Zhang 2011 [49]	53.3/54.5	28/29	Pue (Ivgtt) + Vit B_1_, B_12_	Vit B_1_, B_12_	T2DM (6.5 y)	T2DM (6.8 y)	3 w	①
Song and Xu 2000 [50]	17–82	33/29	Pue (Ivgtt) + Mec (IM)	Vit B_1_, B_12_	Unclear	Unclear	4–8 w	①
Wang 2007 [51]	58.9/59.4	48/46	Pue (Ivgtt) + Mec (Po)	Mec (Po)	T2DM (15.6 y) + DPN (8.9 y)	T2DM (16.2 y) + DPN (8.7 y)	3 m	①②③④⑤
Feng et al. 2004 [52]	58.2/56.3	32/32	Pue (Ivgtt) + Mec (Ivgtt) + Vit B_1_, B_6_ (Ivgtt)	Mec (Ivgtt) + Vit B_1_, B_6_ (Ivgtt)	T2DM (10.3 y) + DPN (4.3 y)	T2DM (11.3 y) + DPN (3.8 y)	4 w	①②③④⑤
Hu and Li 2006 [53]	48	60/40	Pue (Ivgtt)	Vit B_1_, B_12_ (IM)	Unclear	Unclear	2 w	①
Zuo 2004 [54]	56.5	21/22	Pue (Ivgtt)	Danshen (Ivgtt)	Unclear	Unclear	3 m	①③⑤
Bai 2005 [55]	55.3/57.4	50/30	Pue (Ivgtt)	Conventional therapy	DM (11.8 y) + DPN (6.6 y)	DM (12.5 y) + DPN (7.1 y)	4 w	①②③④⑤
Li 2005 [56]	42.9/43.2	60/30	Pue (Ivgtt) + anisodamine (Ivgtt)	Anisodamine (Ivgtt)	DM + DPN (6.7 y)	DM + DPN (7.1 y)	3 w	②③④⑤
Mao 2006 [57]	54	32/20	Pue (Ivgtt) + Mec (IM)	Mec (IM)	Unclear	Unclear	4 w	①②③④⑤
Yang 2016 [58]	54.36/54.1	40/40	Pue (Ivgtt) + Vit B_1_, B_6_ (IM)	Vit B_1_, B_6_ (IM)	DM + DPN (8.24 y)	DM + DPN (8.10 y)	2 m	①
Peng and Ren 2005 [59]	Unclear	60/60	Pue (Ivgtt)	Conventional therapy	DM + DPN (4–16 y)	DM + DPN (4–22 y)	1 m	①④⑤
Dong 2015 [60]	54.18/55.3	40/41	Pue (Ivgtt)	Conventional therapy	Unclear	Unclear	2 m	①②③④⑤

*Note*. T: treatment group; C: control group; y: years; m: months; w: weeks; d: days; DM: diabetes mellitus; T2DM: type 2 diabetes mellitus; DPN: diabetic peripheral neuropathy; Pue: puerarin; Epa: epalrestat; Mec: mecobalamin; Vit B_1_, B_6_, B_12_: vitamins B_1_, B_6_, and B_12_; IV: intravenous infusion; IM: intramuscular injection; Ivgtt: intravenous guttae; Po: per os; ①: the total effective rate; ②: SNCV of median nerve; ③: SNCV of peroneal nerve; ④: MNCV of median nerve; ⑤: MNCV of peroneal nerve.

**Table 2:** Subgroup analysis.

Subgroups	Trials	Effects models	Pooled effect	95% Cl	*P* value
*The total effective rate*					
Pue + Mec versus Mec	11	Fixed	RR 1.31	1.22–1.41	0.00001
Pue + Vit B_1_, B_12_ versus Vit B_1_, B_12_	3	Fixed	RR 1.61	1.24–2.10	0.0004
Pue + Epa versus Epa	2	Fixed	RR 1.38	1.12–1.69	0.002
Pue versus Danshen	5	Fixed	RR 1.44	1.24–1.68	0.00001
*SNCV of median nerve*					
Pue + Mec versus Mec	7	Random	MD 3.64	2.78–4.5	0.0001
Pue + Vit B_1_, B_12_ versus Vit B_1_, B_12_	2	Random	MD 5.43	4.16–6.7	0.00001
Pue + Epa versus Epa	3	Random	MD 3.43	1.28–5.58	0.002
*SNCV of peroneal nerve*					
Pue + Mec versus Mec	8	Random	MD 4.26	2.98–5.55	0.00001
Pue + Vit B_1_, B_12_ versus Vit B_1_, B_12_	3	Random	MD 3.96	2.94–4.97	0.00001
Pue + Epa versus Epa	3	Random	MD 2.57	0.08–5.06	0.04
Pue versus Danshen	2	Random	MD 3.10	1.91–4.29	0.00001
*MNCV of median nerve*					
Pue + Mec versus Mec	7	Random	MD 5.18	3.51–6.85	0.00001
Pue + Vit B_1_, B_12_ versus Vit B_1_, B_12_	3	Random	MD 5.14	2.31–7.97	0.0004
Pue + Epa versus Epa	3	Random	MD 2.32	−0.7–5.34	0.13
*MNCV of peroneal nerve*					
Pue + Mec versus Mec	10	Random	MD 4.54	3.23–5.85	0.00001
Pue + Vit B_1_, B_12_ versus Vit B_1_, B_12_	3	Random	MD 5.01	4.06–5.95	0.00001
Pue + Epa versus Epa	3	Random	MD 2.80	−0.40–6.00	0.09
Pue versus Danshen	2	Random	MD 9.35	−2.13–20.82	0.11

*Note*. Pue: puerarin; Epa: epalrestat; Mec: mecobalamin; Vit B_1_, B_12_: vitamins B_1_ and B_12_; Danshen: danshen injection; MD: weighted mean difference; RR: relative risk.
